# Psychological Factors of Tourist Expenditure: Neglected or Negligible?

**DOI:** 10.3389/fpsyg.2022.942252

**Published:** 2022-06-27

**Authors:** Róbert Štefko, Jozef Džuka, Martin Lačný

**Affiliations:** ^1^Department of Marketing and International Trade, Faculty of Management and Business, University of Prešov, Prešov, Slovakia; ^2^Faculty of Arts, Institute of Psychology, University of Prešov, Prešov, Slovakia; ^3^Faculty of Arts, Institute of Political Science, University of Prešov, Prešov, Slovakia

**Keywords:** tourist expenditure, psychological factors, personality characteristics, willingness to spend, PLS-SEM

## Abstract

Despite recent progress in identifying the factors of tourist expenditure, knowledge of the psychological characteristics of tourists is necessary to fully understand their impact. Therefore, this study attempts to extend the economic, sociodemographic and trip-related factors by including psychological factors in the econometric models. A total of 1,036 Slovak tourists who paid for summer holidays abroad in the summer of 2021 were interviewed. Three of the six psychological factors analysed (two stable personality characteristics – conscientiousness and agreeableness as well as four tendencies expressing willingness to spend or save – spendthrift, tightwad, thrift and spending propensity) correlated significantly with the amount of expenditure. In addition to income, type of travel, children and duration of the stay, the results of the partial least squares test revealed the net effects of tightwad, spending propensity and thrift. The magnitude of the effects of psychological factors points to the need for further research.

## Introduction

Although “…tourists’ spending at destinations around the world is the bread and butter of the tourist economy” (Thrane, 2016, p. 31), some of the related factors have not been sufficiently examined. Regarding their research, Wang and Davidson (2010) have highlighted that the differences in people’s consumer behaviour can explain micro-level analyses better, as macro-level research aggregates travel and expenditure data. Their analysis of existing studies found that income, socio-demographic and trip-related characteristics are the most frequently examined factors of tourist expenditure.^1^ Brida and Scuderi (2013) have reported so-called psychographic variables^2^ in addition to economic, socio-demographic and trip-related factors in their analysis of published studies. There are three groups of variables which can be considered psychographic variables: (a) General opinions and attitudes – simple opinions of respondents regarding various aspects related to the trip such as whether you can have fun, experiencing the outdoors and so on; (b) Opinions on a specific type of trip related to subjective satisfaction with general and specific aspects of the trip such as services and facilities, hospitality, etc.; (c) Motivations in terms of reasons for travel. All three can be considered opinions about situational trip-related characteristics. More recently, Mehran and Olya (2019) published the results of their systematic review and divided the identified factors influencing expenditure into five categories: economic, social, cultural and environmental, psychological and trip-/destination related characteristics. However, like the above-mentioned works in terms of psychological characteristics, their study refers to the lack of data on the psychological characteristics of tourists and the mentioned attempts to model expenditure based on opinions and attitudinal antecedents. In the outlined context their paper highlights the need for future contributions in terms of expenditure modeling and destinations. Alfarhan et al. (2021) have come up with a novel way of testing the differences in expenditure between two groups of people based on their willingness and ability to spend. In this approach, willingness to spend is not defined as a psychological characteristic of a person but rather as a product of the price-quantity composition of international inbound tourism expenditure. The authors tested 11 expenditure factors divided into two groups – socio-economic and trip-specific aspects – with income and length of stay considered the factors with the most consistently reported positive impact on tourism expenditure. The psychological characteristics of tourists were not part of their analysis, and neither were they included by Thrane (2014) who listed 10 similar expenditure factors.

Given that the psychological characteristics of tourists in the tested models of expenditure have not been sufficiently taken into account, the aim of this study was to propose potential psychological factors and verify their effect. In order to achieve the objectives of the study, a survey of 1,036 people was carried out and the data analysed using structural modelling. The following part of this paper is an overview of the related literature and presents the theoretical basis and proposed research model. The research methods and procedures are set out in part three and the findings in part four. The last part summarizes and discusses the theoretical and practical implications and recommendations for future research.

## Theoretical Background and Proposed Research Model

### Usually-Modelled Expenditure Factors

Research into tourist expenditure factors has evolved over the last 20 years, thanks to a ground-breaking article by Marcussen (2011). The result as well as one of the conclusions for further research was that the key variables that explain the variations in tourism expenditure and which must always be part of the analysed variables in the model, are already known. There were ten variables identified: income, age, packaging, travel party size, length of stay, type of accommodation, transportation mode, type of destination, travel distance/nationality and activities. These are considered the major socioeconomic and trip-specific determinants.

#### Income

Brida and Scuderi (2013) have pointed out that income impacted positively and significantly on expenditure in 148 of the 163 studies included in their meta-analysis. Indeed, there were only nine cases which had a negative and statistically significant relationship. The income variable has been found to be significant in most recent research as well (e.g., Correia et al., 2018; Almeida and Garrod, 2021; Alfarhan et al., 2022a).

#### Age

The age of respondents is one of the factors most frequently mentioned when trying to explain tourist expenditure albeit without any general agreement among researchers regarding its possible impact. It has had a positive effect on expenditure in a number of studies with older participants being more inclined to spend than younger ones (Brida and Scuderi, 2013; Pellegrini et al., 2021; Alfarhan et al., 2022a). In contrast, however, some other studies have demonstrated the opposite effect (Bernini and Cracolici, 2015; Yang et al., 2021).

#### Type of Travel

Alfarhan et al. (2022a) have noted that purchasing a tourism package increases the expenditure of high utility-targeting tourists and does not affect the expenditure of low utility-targeting tourists. Travelers who organize their entire trip with tour operators tend to spend more than those who do not make any reservations in advance and only reserve partial elements of the trip (Park et al., 2020).

#### Travel Party Size

The presence of children as a determinant of tourist expenditure has been investigated by numerous authors although no clear relationship has been found (e.g., Wu et al., 2013; Rashidi and Koo, 2016). Pellegrini et al. (2021) reported that the presence of children under 15 contributed to a higher amount of money being allocated to accommodation while everything else was equal.

#### Duration of the Stay

Length of stay is one of the trip specific determinants which has been reported to impact positively on total expenditure (Brida and Scuderi, 2013; Almeida and Garrod, 2021; Gómez-Déniz and Pérez-Rodríguez, 2021; Mortazavi, 2021). Aguiló et al. (2017) have explained that length of stay is the channel through which the effects of all other determinants are translated into total tourist expenditure. It is assumed that the longer the stay, the greater the budget to spend with the positive effect decreasing as the days pass.

#### Gender

Gender has also been included as a sociodemographic dummy variable in the literature with inconclusive results. While some studies have claimed that male tourists spend more than women, other research has shown that female tourists spend more (Aguiló et al., 2017; Gómez-Déniz et al., 2021).

The other four factors usually examined are related to the nature of the destination, the number and type of activities, the mode of transport and purpose of the journey. These have not been included in the current model due to their specific nature (for an overview, see e.g., David-Negre et al., 2018; Park et al., 2020; Alfarhan et al., 2022a). Moreover, Alfarhan et al. (2022b) in their recent approach argue that expenditure and expenditure differentials are, in fact, driven by cognitive differences (namely as regards prosperity at the country of residence and individual travel aspiration), however, in a more aggregated sense.

### Psychological Factors of Expenditure

In their review of micro-analyses of tourist expenditure, Wang and Davidson (2010) concluded that “… theoretically, psychological and destination-related factors may also affect the level of expenditure. Despite this, there has been a limited effort to investigate the role of these variables, presenting a potential area of interest for future research” (p. 521). Ten years later in a review of progress made on outbound tourism expenditure, Mehran and Olya (2019) pointed out that no progress had actually been made in the case of psychological factors. Thus, despite repeated challenges, the psychological characteristics of tourists in relation to expenditure were not tested in models. Recently, Štefko et al. (2020) have made an attempt to search existing literature and propose psychological characteristics. A pragmatic approach was used as a way out of this situation and in an effort to choose psychological variables of expenditure: to look for potential psychological factors of expenditure among the psychological characteristics that are known in the tourism literature and outside the context of expenditure research. The following three psychological characteristics could be found in the analysed literature: personal values, motivation or motive and personality.

In terms of personal values, previous studies (e.g., Li and Cai, 2012) have not used a psychological approach to personal values but a List of Values (LOV, Kahle, 2003) which is not based on a psychological theory of values. Ballantyne et al. (2018) investigated 20 values among visitors to zoos and aquariums. These could be examined separately or combined to reflect 10 basic values following the process outlined by Schwartz et al. (2012): self-direction, stimulation, hedonism, achievement, power, security, conformity, tradition, universalism and benevolence.

As for motivation or motive, Lichy and McLeay (2018) have been critical of the state of understanding of motivation and motives in the tourism literature. Indeed, understanding tourist motivations has been problematic due to the complexity and ambiguity of psychological factors, difficulties in measuring unobservable parameters and the lack of well-developed theory for travel motivation. One example of an often-used theoretical framework or paradigm that underpins many studies of leisure tourism motivation and to which the authors refer, is the push-pull framework. The theory suggests that most tourists travel as a result of being pushed by internal factors and/or being pulled by a set of destination attributes. We would like to make two remarks on this, firstly, that this approach is not sufficiently operationalized and, secondly, that the authors did not base their own approach to finding “generic motivations and types of bleisure travelers” on psychological theory of motivation. It was not about identifying either motivation or motives in the intentions of the psychological understanding of these constructs, but about motivations, motives in terms of reasons for the journey. The integration of motives such as psychological variables of tourist expenditure based on the existing approaches in the field of tourism research appears to be insufficiently prepared both theoretically and methodologically for modelling tourist expenditure factors.

As for personality in the tourism literature, the dominant scientific approach towards personality and its five stable traits can be observed: openness to experience, conscientiousness, extraversion, agreeableness and neuroticism (see Alexander, 2012; Tan and Tang, 2013; Chen et al., 2014; Cohen et al., 2014; Jani, 2014; Passafaro et al., 2015; Kim et al., 2018; Tsiakali, 2018). While it can be assumed that tourists and their personal characteristics are related to tourist behaviour, there does not appear to be any research in which these characteristics and their relationship to expenditure is verified. Soto and John (2017) used the model of the five stable personality traits, also known as the Big Five personality trait domains (e.g., McCrae and Costa, 2008), in developing the first (John et al., 1991) and second version of the Big Five Inventory (BFI-2) questionnaire examining these traits. In the second version, the authors changed the original names of two traits. They used the term Negative Emotionality instead of Neuroticism which has its roots in the term neurosis and expresses the disorder. This emphasizes the domain’s focus on negative emotional experiences rather than the psychiatric disorder. Similarly, they replaced Openness to Experience with the term Open-Mindedness. They wanted to emphasize that it is more about openness to mental experiences and less to social experiences (this is more the content of extraversion). Given the description of these two characteristics, however, it seems unlikely that there would be a relationship between them and summer holiday expenditure. However, the other two seem appropriate – conscientiousness and agreeableness, as pointed out by the conclusions of Soto and John (2017), who analyzed the predictive power of these two domains. Hedonistic behaviour was most strongly predicted by low Conscientiousness and benevolent behaviour by Agreeableness. In order to support the suitability of these two personality traits, Hengartner et al. (2020) verified the stability of all five traits surveyed by the first version of the BFI questionnaire (John et al., 1991). They found that Conscientiousness and Agreeableness showed the greatest stability compared to the other three traits.

While the relationship between stable personality traits and expenditure is unclear, other personal characteristics can be assumed to be related to the level of expenditure. These are characteristics known from research on consumer behaviour and are psychological characteristics expressing the willingness or unwillingness to pay. Rick et al. (2008) have previously published the idea of two different ways of experiencing a situation in which a person has to pay: “We propose that an anticipatory pain of paying drives ‘tightwads’ to spend less than they would ideally like to spend. ‘Spendthrifts,’ by contrast, experience too little pain of paying and typically spend more than they would ideally like to spend” (p. 767). They consider both characteristics to be the basis for individual differences in willingness to pay (in their terms “in the pain of paying”) and believe that these individual differences are likely to have important consequences for behaviour. They have developed a short tool for assessing both inclinations which they believe can reveal these individual differences through indirectly worded questions. Given that two of the instrument’s four questions are formulated as relatively comprehensive scenarios describing the behaviour of two shoppers, they do not seem appropriate for a retrospective self-assessment of this subjective tendency. Therefore, only the remaining two formulations from the original tool have been adapted for the current research.

A multi-thematic approach measuring spending propensity has been found in the current literature to support this aspect of measuring tourist expenditure based on individual differences in willingness to spend. Chandrashekaran (2019) has used a scale of 4 items to measure this inclination with the total score being interpreted as follows: “Rather, spending propensity is an intrinsic characteristic that influences willingness to pay across a wide range of purchase situations, regardless of price” (p. 196). Although the characteristic of a person defined in this way was examined in conditions of a specific experimental situation, it is possible to investigate it using a questionnaire retrospectively as a subjectively evaluated disposition.

According to the analysed literature, the measurement of spending propensity can be based on two procedures, the one-item formulation by Rick et al. (2008; “Spendthrifts”) and the four-item scale by Chandrashekaran (2019). We believe that it is appropriate to supplement the characteristic “Tightwads” with a multiitem tool as well. Such an approach is known in the literature and is associated with the term “frugality” or thrift. Lastovicka et al. (1999) were first to do empirical research on frugality and their findings point out that frugality consistently explains consumer usage behaviours and that being frugal empirically affects purchasing. The authors have developed a scale to measure frugality and state that it is a reliable tool (Cronbach’s alpha of the eight-item sum scale in the data was 0.85 or in the general population 0.80).

### Proposed Research Model

**FIGURE 1:**
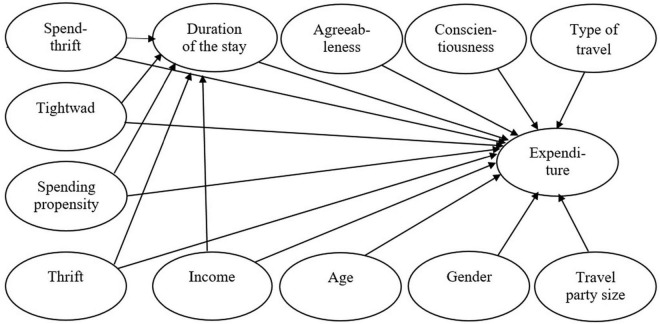
Path model under cosideration.

The model aimed at explaining variance in tourism expenditure should incorporate most of the confirmed independent variables from previous research (compare Alfarhan et al., 2021) as well as adding a few extra. Indeed, the explained variance in expenditure should not be less than around 30% (Thrane, 2014). The following six variables were considered to be important for the current research: income, age, type of travel, travel party size, gender and duration of the stay. While the models tested so far have not included psychological factors, six of the twelve independent variables in the present model design (Figure 1) represent the psychological characteristics of tourists. These are two stable personality traits – agreeableness and conscientiousness as well as four other personal traits that represent willingness/unwillingness to pay. In addition to the direct relationship between these four personal characteristics, the mediation effect of duration of the stay was tested due to the important position of this variable (see Aguiló et al., 2017). It was assumed that spendthrift and spending propensity would be positively related to duration of the stay and affect expenditure positively while tightwad and thrift would be negatively related to the duration of the stay and affect expenditure negatively. To the best of our knowledge, there have been no research findings published on the relationship of agreeableness and conscientiousness with expenditure. Therefore, the direction of the relationship between these variables and expenditure has not been determined.

## Methodology

### Measurement

The electronically administered questionnaire consisted of three parts. The first part included questions related to the trip. “Were you on holiday abroad during the summer of 2021? By foreign holiday we mean a summer holiday stay for the purpose of recreation lasting at least 5 nights, which you spent in the months of July and August. You could have travelled abroad as early as June or you could have returned in September as well.” Only those who answered “yes” continued. Note: In determining the lower limit of the number of overnight stays, we relied on statistical data concerning the usual length of Slovak summer holidays and on the dominant types of summer holiday stays sold by domestic travel agencies. Less frequent types of summer holidays lasting less than 5 nights were not included in the analysis.

Type of travel identified whether the holiday stay was organized individually (0) or purchased as a package through a travel agency (1).

Travel party size identified the number of traveling adults and number of traveling children.

Duration of the stay (number of overnight stays) was reported numerically.

Expenditure was detected by the question: “What was the total amount in euros you spent on your holiday – it means all the expenses associated with the holiday stay (if you were two or a family – the total amount for all).”

In the second part of the questionnaire, there were questions representing six psychological constructs.

Twenty-four items from the Big Five Inventory (BFI-2, Soto and John, 2017) were extracted to measure two stable personality traits – agreeableness and conscientiousness.

Spending Propensity (Chandrashekaran, 2019) was assessed using four items – two items related to the inclination to spend freely and the remaining two items corresponded to the proclivity to curb spending. The two items corresponding to limiting spending were reverse coded so that the larger numbers represented higher spending propensity. The participants’ Spending Propensity score was calculated as the sum of the ratings on the four items. For each item, the participants indicated their agreement on a 6-point scale ranging from Completely Disagree to Completely Agree.

There were six items measuring thrift (unpublished). The items of this thrift scale were developed independently by the second author of the current study and are comparable to the items of the frugality scale developed by Lastovicka et al. (1999).

Tightwad and spendthrift are 2 items whose wording has been adapted from the four-item tightwad-spendthrift scale (TW-ST, Rick et al., 2008). These two items focus on whether consumers have difficulty controlling their spending or difficulty forcing themselves to spend. These two one-item questions are not expected to be identical in content to the previously mentioned two multi-item scales. As these did not measure endogenous target constructs, their inclusion in the analysis can be considered non-problematic in this respect.

The participants responded to 24 items measuring Agreeableness and Conscientiousness as well as four items of the spending propensity scale, six items of the thrift scale and one item for tightwad and one for spendthrift on a five-point scale from not suitable for me to completely suitable for me (quite like me); (more details on this can be found in the Appendix Table A in Supplementary Material).

The items for measuring the demographic characteristics of tourists were in the third part of the questionnaire: gender, age, marital status (single, married, other) and education (primary education, secondary without A-Levels, secondary with A-Levels and university). Income was identified by the last question in the questionnaire. The respondents reported the average net personal monthly income for the last 12 months so that they could classify it into one of 8 categories: from less than 500, 501 – 750 euros to more than 2,001 euros. They could also use two other options for this question: I don’t have my own income or I don’t want to state it.

### Data and Procedure

The data were collected through an agency. The data collection spanned about 3 weeks during September and October 2021. The selection of the sample ensured representation of people from all over the country – each of the eight self-governing regions of Slovakia (population 5,458,000) was represented by a proportional number of interviewed people (range 90–175 people). In addition to the proportional representation of the regions, a quota selection was also applied in relation to age. By this, persons of three age categories (25–40, 41–55 and 56–70 years) were proportionally represented in the sample. There were no quotas used to select persons in terms of their economic activity. The respondents had online access to the questionnaire *via* an e-mail from the agency. The initial sample consisted of 1,036 participants and 820 participants (79.2%) were finally obtained after excluding invalid questionnaires (they were excluded if participants had marked the answers to the question about personal monthly income as “I do not have my own income” or “I do not want to state”). In terms of procedure, respondents were assured of the anonymity of the survey: “The information obtained will be for statistical purposes only, will not be associated with you in any way and will only be processed in bulk with the answers of other survey participants.” Demographic data on the analyzed sample are in Table 1.

**TABLE 1:** Sociodemographic characteristics of the respondents (*N* = 820).

	Frequency	Percentage
**Gender**		
Women	459	56
Men	361	44
**Age**		
25–40	381	46.5
41–55	302	36.8
56–70	137	16.7
**Marital status**		
Married	466	56.8
Single	260	31.7
Other	94	11.5
**Education**		
Primary education	2	0.2
Secondary education without A-Levels	58	7.1
Secondary education with A-Levels	326	39.8
University education	434	52.9
**Average net personal monthly income**		
less than 500€	49	6
501€ to 750€	144	17.6
751€ to 1,000€	218	26.5
1,001€ to 1,250€	164	20
1,251€ to 1,500€	113	13.8
1,501€ to 1,750€	56	6.8
1,751€ to 2,000€	27	3.3
Over 2,001€	49	6

### Analytical Approach

The data analysis was performed in three steps. First, the reliability of the four multi-item scales – Agreeableness, Conscientiousness, Spending Propensity and Thrift – was verified. A descriptive and correlation analysis of all the variables was then performed. These analyses were performed using IBM SPSS Statistics 22. In the third step, an analysis of the complex relationships in the conceptual model was performed using PLS-SEM. The choice of data analysis resulted from the research objective which was to assess the PLS path model’s explanatory power. This is in line with the recommendations of Hair et al. (2022, p. 31) who claim “…if the primary research objective is prediction and explanation of target constructs (Rigdon, 2012), PLS-SEM should be given preference (Hair et al., 2017, 2019).” The PLSpredict (Shmueli et al., 2016) was used to assess the PLS path model’s predictive power. SmartPLS 3.3.7 software was used and the study complied with the recommendations of Sarstedt et al. (2022) in presenting the results.

**FIGURE 2:**
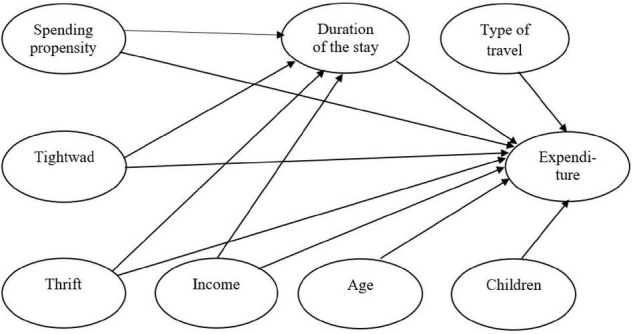
Tested conceptual model.

Figure 2 shows the tested conceptual model. The predicted variable was the amount of expenditure while the explanatory variables were income (ordinal variable), type of travel (binary variable), number of traveling children, duration of the stay and three psychological variables – two multi-item scales – spending propensity and thrift and one one-item question – tightwad. The effect of age was controlled. In the case of duration of the stay, the mediation effect was tested in addition to the direct effect: length of stay as a mediator of the influence of three psychological variables on the amount of expenditure. In comparison to the original design of the conceptual model (Figure 1), variables whose correlations with expenditure were insignificant were omitted from the tested model: gender and number of adult travellers (0.050, or 0.018) and three other originally intended psychological variables, agreeableness, conscientiousness and spendthrift (–0.024, 0.019, or 0.043). (The correlation matrix of all the variables is in the Appendix Table B in Supplementary Material).

## Results

### Descriptive Statistics and Correlations

Table 2 shows the descriptive statistics and correlations of the eight analysed expenditure factors. All eight factors correlate with the level of expenditure in the expected direction: higher income, holiday stay purchased as a package, length of stay, number of children and spending propensity correlated positively while tightwad and thrift correlated negatively. The effect of age as a controlled variable was negative and higher age represented lower expenditure.

**TABLE 2:** Means, standard deviations, and Pearson correlations.

Variables	*M*	SD	2	3	4	5	6	7	8	9
1. Expenditure	1,389	886	0.23**	0.30**	–0.09*	0.31**	0.24**	–0.11**	0.09**	–0.12**
2. Income	3.82	1.77		0.03	–0.12**	0.04	0.06	–0.01	0.04	–0.12**
3. Type of travel	0.40	0.50			–0.07*	–0.01	0.08*	0.10**	–0.02	0.04
4. Age	43.00	11.75				0.05	–0.12**	–0.09**	–0.19**	–0.08*
5. Duration	7.80	2.50					–0.01	–0.10**	–0.04	–0.05
6. Children	0.62	0.87						0.01	–0.04	0.06
7. Tightwad	1.87	0.99							–0.16**	0.41**
8. Spending p.	3.04	0.91								–0.28**
9. Thrift	2.53	0.87								

*M and SD are used to represent the mean and standard deviation, respectively.*

*Indicates p < 0.05. **Indicates p < 0.01.

### Assessment of the Measurement Models

Table 3 shows the values of convergent validity of both scales (AVE value), values of the loading indicators which constitute both scales, and the values of reliability of both scales (Cronbach’s alpha, rhoA and rhoC).

**TABLE 3:** Assessment of convergent validity and internal consistency reliability.

Scala/items	Loading	Cronbach’s alpha	rhoA	rhoC	AVE
*Spending propensity*		0.713	0.706	0.815	0.527
Spendp1	0.651				
Spendp2	0.660				
Spendp3r	0.775				
Spendp4r	0.806				
*Thrift*		0.829	0.865	0.870	0.528
Thrift1	0.756				
Thrift2	0.720				
Thrift3	0.776				
Thrift4	0.747				
Thrift5	0.660				
Thrift6	0.696				

The results show that two of the reflectively measured constructs’ measures, spending propensity and the Thrift scale, which has two items less than a comparable tool (Lastovicka et al., 1999), are reliable and valid (Table 3). Although not all loadings of the tools exceed the threshold value of 0.708, the average variance extracted (AVE) is higher than the critical value of 0.5, and all the construct reliabilities i.e., Cronbach’s alpha, the coefficients rhoA, and the composite reliability rhoC have values above 0.7 (Sarstedt et al., 2022).

The discriminant validity assessment, based on the heterotrait-monotrait (HTMT) ratio of correlations measure (Henseler et al., 2015), shows that all the HTMT values are significantly lower than 0.85, thus supporting the measures’ discriminant validity (Table 4).

**TABLE 4:** Assessment of discriminant validity using the heterotrait-monotrait ratio of correlations criterion.

Variables	1	2	3	4	5	6	7	8	9
1. Age									
2. Children	0.116								
3. Duration	0.047	0.006							
4. Expenditure	0.089	0.244	0.313						
5. Income	0.119	0.059	0.040	0.225					
6. Spending p.	0.225	0.042	0.050	0.109	0.044				
7. Thrift	0.091	0.063	0.064	0.135	0.128	0.371			
8. Tightwad	0.093	0.005	0.097	0.112	0.007	0.188	0.447		
9. Type of travel	0.073	0.081	0.008	0.295	0.025	0.047	0.049	0.102	

### Structural Equation Modelling Analysis

#### Collinearity

The structural model was firstly assessed for collinearity issues by examining the variance inflation factor (VIF) values of all the predictor constructs in the model. Table 4 shows the VIF values of all predictors in the model. All values are less than 2 which indicates that collinearity is not a problem.

#### Significance and Relevance of the Path Coefficients (Standardized Regression Coefficients)

**FIGURE 3:**
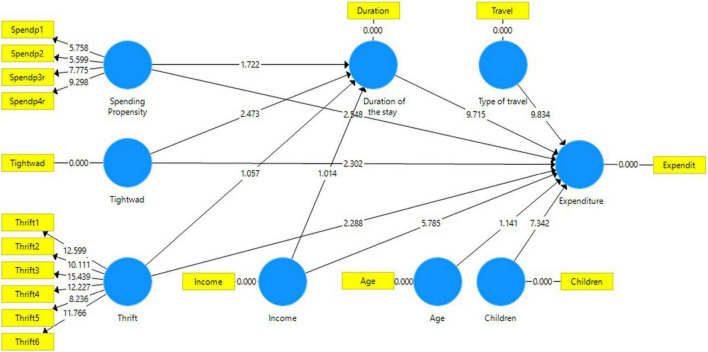
Tested model and the estimation. Bootstrap t-statistics based on 820 cases and 5,000 replications.

The results of the bootstrapping procedure with 5,000 samples has revealed (Figure 3 and Table 4) that all path coefficients of the tested expenditure factors are significant with the exception of age [–0.034, (*p* > 0.10)]. In particular, the effects of three psychological variables, tightwad –0.073 (*p* < 0.05), spending propensity 0.078 (*p* < 0.01) and thrift –0.071 (*p* < 0.05) and four expenditure factors – income 0.177 (*p* < 0.01), type of travel 0.285 (*p* < 0.01), children 0.214 (*p* < 0.01) and duration of the stay 0.303 (*p* < 0.01) were in line with expectations. With regards to the tested mediation of the three psychological factors, only tightwad –0.091 (*p* < 0.05) had a significant relationship with duration of the stay and the specific indirect effect –0.028 (*p* < 0.05) was significant as well.

In addition to the significant path coefficients, Table 5 also presents the values of the effect size of the tested variables *f*^2^. The guidelines for assessing *f*^2^ values are that values of 0.02, 0.15, and 0.35 represent small, medium, and large effects of the corresponding exogenous variables (Cohen, 1988). The psychological factors in the current structural model do not have a significant effect on expenditure and the four significant exogenous variables – income (*f*^2^ = 0.043), type of travel (*f*^2^ = 0.114), duration of the stay (*f*^2^ = 0.129) and children (*f*^2^ = 0.064) have less than a medium effect on expenditure.

**TABLE 5:** Assessment of the structural model.

Reationship	Std beta	*p*-value	VIF	*f* ^2^	*R* ^2^	*Q* ^2^
Spending p. → Expenditure	0.078	0.010	1.179	0.007	0.300	0.194
Tightwad → Expenditure	–0.073	0.024	1.229	0.006		
Thrift → Expenditure	–0.071	0.022	1.337	0.005		
Income → Expenditure	0.177	0.000	1.040	0.043		
Age → Expenditure	–0.034	0.255	1.105	0.001		
Children → Expenditure	0.214	0.000	1.026	0.064		
Type of travel → Expenditure	0.285	0.000	1.021	0.114		
Duration → Expenditure	0.303	0.000	1.018	0.129		
Spending p. → Duration	–0.077	0.091	1.107	0.005	0.017	0.005
Tightwad → Duration	–0.091	0.015	1.313	0.007		
Thrift → Duration	–0.045	0.292	1.204	0.002		
Income → Duration	0.037	0.306	1.018	0.001		

#### In-Sample Model Fit and Explanatory Power

Table 5 shows the *R*^2^ values for the endogenous constructs. The *R*^2^ value for expenditure (0.30) confirms that the tested model was specified correctly. The value for duration of the stay is very low (0.02).

#### Out-Of-Sample Predictive Power

PLSpredict was used with 10 folds and 10 repetitions to mimic how the PLS model would eventually be used to predict a new observation. As can be seen from Table 5, the *Q*^2^ value in the case of the expenditure prediction is 0.19. At the same time, the root mean squared error (RMSE) generated by the PLS-SEM based estimates was compared with those of a linear benchmark model (Shmueli et al., 2019). The expenditure RMSE values are lower for the PLS-SEM than for the Linear Model (798.027, or 796.366). Based on this, it can be concluded that the PLS path model has a predictive power for expenditure.

## Discussion, Conclusions, and Implications

Three of the four tested factors representing the psychological characteristics of tourists had a significant relationship with the amount of expenditure, the path-coefficient value of spendthrift was insignificant. Another significant factors were income, stay purchased as a package, number of traveling children and length of stay, the path-coefficient values of age and gender were insignificant. Regarding the indirect effect, only tightwad from the personal characteristics tested in the model expressing willingness/unwillingness to pay had a significant relationship with the amount of expenditure. This was found to be mediated by the length of the stay.

In line with previous research, income has been confirmed as a significant economic factor of tourist expenditure (e.g., Correia et al., 2018; Almeida and Garrod, 2021; Alfarhan et al., 2022a). Similarly, the purchase of a holiday as a package involves higher expenditure than a holiday organized by the tourist himself (e.g., Park et al., 2020). In terms of travel party size, the number of traveling children has not been clearly demonstrated as an expenditure factor in previous studies (e.g., Wu et al., 2013; Rashidi and Koo, 2016). However, the present results are consistent with the findings of Pellegrini et al. (2021) who have stated that the presence of children aged below 15 years contributed to a higher amount of money allocated to accommodation. While the present analysis did not look specifically at where the higher expenditure was allocated, the effect of the number of traveling children was significant. The length of stay also had a comparable relationship to expenditure like the purchase of a holiday as a package (compare Almeida and Garrod, 2021; Mortazavi, 2021; Gómez-Déniz and Pérez-Rodríguez, 2021). Therefore, three travel-related factors, the purchase of a holiday as a package, the travel party size and the duration of the stay, can be considered significant expenditure factors. The two socio-demographic factors tested did not have a significant relationship to expenditure which is in line with previous findings stating that the effect of age and gender is ambiguous. In the case of age e.g., Alfarhan et al. (2022a) has found that the elderly tourists spent more while Yang et al. (2021) has found that younger tourists had higher expenditue. Likewise, contradictory findings have also been identified in the case of gender (compare Saayman and Saayman, 2012; Aguiló et al., 2017, or Gómez-Déniz et al., 2021).

The discriminant validity values of all measures in the tested structural model met the required criteria. This means that each construct is empirically unique and captures a phenomenon that other constructs in the PLS path model do not represent (Franke and Sarstedt, 2019). The seven statistically significant expenditure factors tested explained 30% of the variance in expenditure. This indicates a sufficient model’s explanatory power (Shmueli, 2010) and can be considered a value that allows the model to be assessed as adequately specified (Thrane, 2014). At the same time, it can be stated that the model does not only have explanatory but also predictive power in relation to expenditure (*Q*^2^ = 0.194; Shmueli et al., 2016). In terms of the effect size, however, the four significant exogenous variables – income (*f*^2^ = 0.043), type of travel (*f*^2^ = 0.114), presence of children (*f*^2^ = 0.064) and duration of the stay (*f*^2^ = 0.129) have not reached the limit of medium effect size on expenditure. Regarding psychological characteristics, three out of four psychological factors in the tested conceptual model – spending propensity, tightwad and thrift (spendshrift, agreeableness and conscioutness, which were assumed in the original model design, did not correlate with expenditure, therefore they were not included in the tested model), did not achieve even small effect. The design of a tourist expenditure model was probably one of the first attempts to take into account those psychological factors that have not been examined in tourist expenditure models to date. However, this initial idea was not comprehensive enough, and as the unknown reviewer pointed out, the interrelationships between the psychological factors themselves remained unrecognized. In particular, the proposed psychological factors represent two different types of psychological characteristics of persons – relatively stable personality traits and spending habits which are probably more dependent on external factors. In particular, it is likely, for example, that conscientiousness as a relatively stable personality characteristic may not be directly related to the amount of expenditure, as shown in our analysis, but may be related to expenditure behaviour. Therefore, conscientiousness may not affect the amount of expenditure directly, but mediated through some of the variables of expenditure behaviour (eg, thrift, tightwad). Furthermore, while relatively stable characteristics, in our research conscientiousness and agreeableness, are less dependent on demographic variables (gender, age, education, etc.), spending habits, in our research thrift and tightwad can be moderated by these variables. This consideration at this stage is rather speculative, but further research in this regard could provide a basis for a better understanding of the psychological factors in tourist expenditure.

Moreover, we tried to verify the relationship of the three psychological factors to the amount of expenditure and the length of stay separately, as did Aguiló et al. (2017) in their research of usually examined determinants of tourist expenditure, but in contrast, we tested the effect of the length of stay as a mediator. The value of only one significant path-coefficient expressing the specific indirect effect of tightwad (–0.028), length of stay and expenditure did not represent a meaningful size of the mediation effect. Therefore, while psychological factors have been neglected in previous tourist expenditure studies, the results of the current research have indicated that they are negligible in terms of their impact on expenditure. In the next section, this finding will be interpreted in the context of the theoretical and practical implications.

### Theoretical Contributions

Given that the tested model explained the expected percentage of expenditure variance supports the correctness of the model specification both in terms of the choice of dependent variable and chosen independent variables. The theoretical contribution of the obtained results to the econometric modelling of tourism expenditure can be seen on two levels. First, stable personality traits such as agreeableness and conscientiousness, are unrelated to summer holiday expenditure. Indeed, it remains to be seen whether this type of psychological traits should be taken into account in future tourist expenditure research. Second, three out of the four psychological factors – spending propensity, tightwad and thrift, expressing a person’s overall willingness/unwillingness to spend, can be preliminarily considered a contribution to the econometric modelling of tourist expenditure. They must be considered preliminarily because firstly, this is the first research to the best of our knowledge verifying their relationship to expenditure and secondly, their effect size is insufficient. There are three explanations for the insufficient size of the identified effects. First, spending propensity, tightwad and thrift have been investigated in this research as general characteristics of individuals. These may manifest themselves in different situations when individuals are confronted with the realization of expenditure. However, there was not a specific manifestation of these characteristics in relation to spending or saving on a summer holiday. Indeed, the wording of the items does not express the context of a summer holiday. The proposal to reformulate the wording of the measures so that spending or saving relates to a summer holiday situation can be considered an appropriate theoretical contribution to further research into the psychological characteristics of people as a factor in tourist expenditure. Second, summer holidays represent an outlay that can be perceived as a one-off expense on “luxury goods.” Therefore, the usual person-specific setting in terms of the tendency to spend or save may not be apparent in this case. In other words, people react less predictably to this type of outlay – for example, those who usually save may spend more on holiday. There is no support for this consideration in the analysis, however, and this explanation may be nothing more than speculation. A third possible explanation is the fact that the three psychological characteristics identified are not really factors in tourist expenditure, and therefore their inclusion in econometric models of expenditure may be unjustified.

### Practical Implications

The practical usefulness of the tested model as a whole and the identified variables that predict the amount of expenditure is indicated by the result of the model’s predictive ability test (Sarstedt and Danks, 2021). The three short tools for determining psychological characteristics, two scales of spending propensity and thrift and one-item evaluation of tightwad identify antecedents that are significantly related to the amount of expenditure. In particular, these are the expenses that represent the total amount that a person spends on a summer holiday. In other words, all the expenses associated with a holiday stay. The research use of these tools is a second practical implication due to their very good reliability, convergent and divergent validity. The practical use of these tools in the field of tourism management for recognizing the characteristics of summer holiday buyers is not yet possible due to the insufficient size of their effects.

### Limitations and Paths for Future Research

We would like to divide the limitations into two groups: the first group of limitations is related to non-psychological variables (thanks to an unknown reviewer for notifying us), the second group is related to the choice of psychological variables of tourist expenditure. It can be considered as a limitation that in our analysis we did not include the independent variable “mode of transport” in the tested model of total expenditure, which could result in uncontrolled heterogeneity in the distribution of tourists expenditure. Furthermore, we did not take into account the buyer’s decision-making processes from the utility maximization/expenditure minimization aspect. Finally, in the tested model we investigated the effect of duration of the stay on expenditure, but did not consider the potential problem of endogeneity of length of stay (see Thrane, 2015; Aguiló et al., 2017; Alfarhan et al., 2022a). We tested the length of stay in the model as a mediator of the relationship between psychological variables and the amount of expenditure, and therefore it remains to take into account the mentioned aspect of the variable length of stay in future analyzes. The choice of psychological variables was motivated by the fact that the model of expenditure factors took into account the psychological characteristics which were already known and had been published in tourism research. While this was an advantage to a certain extent given the availability of existing knowledge on psychological variables in the tourism literature, one limitation was that other potential psychological expenditure factors have not been taken into account. For example, extraversion or neuroticism can be considered from stable psychological characteristics. Hence, a still valid requirement to take into account psychological variables in econometric models reflected by researchers for several years (Wang and Davidson, 2010; Brida and Scuderi, 2013; Mehran and Olya, 2019 and others), remains a challenge also for future research. This challenge can be addressed by paying attention to other characteristics which are the subject of psychological science research and which could be expected to be related to the expenditure behaviour of tourists. The second recommendation for future research is a direct follow-up to the results of this research. It is a change in the approach to measuring the three psychological characteristics – spending propensity, thrift and a tightwad. Future research might try not to measure spending propensity, thrift and tightwad as general characteristics which may manifest themselves in various situations in which persons are confronted with expenditure, but reformulate the wording of the items so that spending or saving represents a specific situation of a summer holiday.

## Data Availability Statement

The raw data supporting the conclusions of this article will be made available by the authors, without undue reservation.

## Ethics Statement

Ethical review and approval was not required for the study on human participants in accordance with the local legislation and institutional requirements. Written informed consent for participation was not required for this study in accordance with the national legislation and the institutional requirements.

## Author Contributions

RŠ: conceptualization and methodology (tourism marketing). JD: conceptualization and methodology (psychological aspects and selection of psychological tools), Software, data curation, writing – original draft preparation and reviewing and editing. ML: conceptualization and methodology (economic, sociological, and political aspects), literature review, writing – original draft preparation and reviewing and editing. All authors contributed to the article and approved the submitted version.

## Conflict of Interest

The authors declare that the research was conducted in the absence of any commercial or financial relationships that could be construed as a potential conflict of interest.

## Publisher’s Note

All claims expressed in this article are solely those of the authors and do not necessarily represent those of their affiliated organizations, or those of the publisher, the editors and the reviewers. Any product that may be evaluated in this article, or claim that may be made by its manufacturer, is not guaranteed or endorsed by the publisher.

## Footnotes

1 We use the term “tourist expenditure” in the sense of individual summer holiday expenses. Alternatively, due to the economy of expression, the term “expenditure” is used.

2 The term “psychographic” is not defined in psychology.
