# Supplementary material for: Psychological Factors of Tourist Expenditure: Neglected or Negligible?
Source: Front Psychol. 2022 Jun 27;13:942252. doi: 10.3389/fpsyg.2022.942252 (PMC9271940; doi:10.3389/fpsyg.2022.942252)
Supplement: Supplementary file 1 [file Data_Sheet_1.docx]

Supplementary Material

# Table A: Supplementary Data

| Constructs | Source | Items |
| --- | --- | --- |
| Spending Propensity | Chandrashekaran (2019) | Spendp1: I spend money quite freely |
|  |  | Spendp2: Spending money makes me happy |
|  |  | Spendp3r: I am very careful with what I spend my money on |
|  |  | Spendp4r: I will spend money on something only if I really need to |
| Thrift | Džuka (unpublished) | Thrift1: I almost always hesitate before I buy something for myself. |
|  |  | Thrif2: When I buy clothes, I think, "What is it for? I still have old ones." |
|  |  | Thrift3: I feel anxious when I have to spend money. |
|  |  | Thrift4: I always try to shop as cheaply as possible. |
|  |  | Thrift5: I'm nervous when I spend more money than I planned. |
|  |  | Thrift6: If an older appliance breaks down, I try to repair it so that I don't have to spend money on a new one. |
| Tightwad and Spendthrift | Rick, Cryder, & Loewenstein (2008) | Tightwad: Some people have a problem limiting how much money they spend, even though they know they shouldn't spend so much.  Mark one answer as appropriate for you. |
|  |  | Spendsh: Some people find it difficult to pay (spend money) and often fail to do so, even though they know they should.  Mark one answer as appropriate for you. |
| Agreeableness,  Conscientiousness | Soto & John (2017) | Agree1: Is compassionate, has a soft heart |
|  |  | Agree2: Is respectful, treats others with respect |
|  |  | Agree3r: Tends to find fault with others |
|  |  | Agree4r: Feels little sympathy for others |
|  |  | Agree5r: Starts arguments with others |
|  |  | Agree6: Has a forgiving nature |
|  |  | Agree7: Is helpful and unselfish with others |
|  |  | Agree8r: Is sometimes rude to others |
|  |  | Agree9r: Is suspicious of others’ intentions |
|  |  | Agree10r: Can be cold and uncaring |
|  |  | Agree11: Is polite, courteous to others |
|  |  | Agree12: Assumes the best about people |
|  |  | Consc1r: Tends to be disorganized |
|  |  | Consc2r: Tends to be lazy |
|  |  | Consc3: Is dependable, steady |
|  |  | Consc4: Is systematic, likes to keep things in order |
|  |  | Consc5r: Has difficulty getting started on tasks |
|  |  | Consc6r: Can be somewhat careless |
|  |  | Consc7: Keeps things neat and tidy |
|  |  | Consc8: Is efficient, gets things done |
|  |  | Consc9: Is reliable, can always be counted on |
|  |  | Consc10r: Leaves a mess, doesn’t clean up |
|  |  | Consc11: Is persistent, works until the task is finished |
|  |  | Consc12r: Sometimes behaves irresponsibly |

# Table B: Correlations of the all variables

|  | 2 | 3 | 4 | 5 | 6 | 7 | 8 | 9 | 10 | 11 | 12 | 13 | 14 |
| --- | --- | --- | --- | --- | --- | --- | --- | --- | --- | --- | --- | --- | --- |
| 1. Expenditure | .225** | .295** | .050 | -.089* | .313** | .018 | .244** | -.024 | -.112** | .019 | .043 | .091** | -.123** |
| 2. Income |  | .025 | -.195** | -.119** | .040 | -.019 | .059 | -.044 | -.007 | -.042 | .036 | .035 | -.117** |
| 3. Type of travel |  |  | -.040 | -.073* | -.008 | .047 | .081* | .077* | .102** | -.026 | -.041 | -.021 | .040 |
| 4. Gender |  |  |  | .006 | .024 | -.070* | -.020 | .031 | -.022 | .157** | .121** | .072* | -.035 |
| 5. Age |  |  |  |  | .047 | -.020 | -.116^**^ | -.129^**^ | -.093^**^ | .188^**^ | .132^**^ | -.190^**^ | -.081^*^ |
| 6. Duration |  |  |  |  |  | .015 | -.006 | -.047 | -.097^**^ | .078^*^ | .075^*^ | -.042 | -.052 |
| 7. Adult |  |  |  |  |  |  | .000 | .000 | -.006 | .019 | .001 | -.017 | .027 |
| 8. Children |  |  |  |  |  |  |  | .019 | .005 | -.034 | -.055 | -.036 | .056 |
| 9. Spendthrift |  |  |  |  |  |  |  |  | .248^**^ | -.178^**^ | -.243^**^ | .352^**^ | .115^**^ |
| 10. Tightwad |  |  |  |  |  |  |  |  |  | -.200^**^ | -.150^**^ | -.157^**^ | .405^**^ |
| 11. Agreeableness |  |  |  |  |  |  |  |  |  |  | .535^**^ | -.144^**^ | -.115^**^ |
| 12. Conscientiousness |  |  |  |  |  |  |  |  |  |  |  | -.294^**^ | -.056 |
| 13. Spending propensity |  |  |  |  |  |  |  |  |  |  |  |  | -.282^**^ |
| 14. Thrift |  |  |  |  |  |  |  |  |  |  |  |  |  |

*Note.* * indicates *p* < .05. ** indicates *p* < .01.
